# Viscosity dynamics and the production of extracellular polymeric substances and soluble microbial products during anaerobic digestion of pulp and paper mill wastewater sludges

**DOI:** 10.1007/s00449-019-02224-4

**Published:** 2019-10-10

**Authors:** Eva-Maria Ekstrand, Bo H. Svensson, Luka Šafarič, Annika Björn

**Affiliations:** Department of Thematic Studies, Environmental Change, Linköping University, 581 83 Linköping, Sweden

**Keywords:** Rheology, Extracellular polymeric substances, Soluble microbial products, OLR, Sludge bulking

## Abstract

The production processes of the pulp and paper industry often run in campaigns, leading to large variations in the composition of wastewaters and waste sludges. During anaerobic digestion (AD) of these wastes, the viscosity or the production of extracellular polymeric substances (EPS) and soluble microbial products (SMP) may be affected, with the risk of foam formation, inefficient digester mixing or poor sludge dewaterability. The aim of this study was to investigate how viscosity and production of EPS and SMP during long-term AD of pulp and paper mill sludge is affected by changes in organic loading rate (OLR) and hydraulic retention time (HRT). Two mesophilic lab-scale continuous stirred tank reactors (CSTRs) were operated for 800 days (R1 and R2), initially digesting only fibre sludge, then co-digesting fibre sludge and activated sludge. The HRT was lowered, followed by an increase in the OLR. Reactor fluids were sampled once a month for rheological characterization and analysis of EPS and SMP. The production of the protein fraction of SMP was positively correlated to the OLR, implicating reduced effluent qualities at high OLR. EPS formation correlated with the magnesium content, and during sulphate deficiency, the production of EPS and SMP increased. At high levels of EPS and SMP, there was an increase in viscosity of the anaerobic sludges, and dewatering efficiency was reduced. In addition, increased viscosity and/or the production of EPS and SMP were important factors in sludge bulking and foam formation in the CSTRs. Sludge bulking was avoided by more frequent stirring.

**Electronic supplementary material:**

The online version of this article (10.1007/s00449-019-02224-4) contains supplementary material, which is available to authorized users.

## Introduction

Rheological properties of sludge (e.g., viscosity) affect several important parameters in waste treatment processes, such as pumping, mixing and sludge dewatering [1, 2]. More specifically, increased viscosity during anaerobic digestion (AD) may negatively affect mixing efficiency, leading to dead zones and decreased process performance [3]. Most research on the factors affecting sludge rheology so far addressed activated sludge, primarily showing a positive correlation between viscosity and the amount of total solids (TS) [4–7]. For AD sludges, however, additional regulators for sludge viscosity have been identified, such as temperature [8], substrate type [9] and hydraulic retention time (HRT) [10]. At pulp and paper mills, the production processes are often run in campaigns to meet product demands, i.e., switching between bleached and unbleached pulp production and using different raw materials. This leads to variations in the composition of wastewaters and waste sludges (i.e., fibre sludge, activated sludge), which, when used as substrates for AD, affect the microbiological treatment processes and likely alters the rheological properties of the AD sludge. Furthermore, rheological properties of fibre suspensions have been well studied [11, 12], showing that rheology is affected by factors such as the concentration of fibres in the liquid or fibre length. In addition, AD of waste-activated sludge (WAS) from the pulp and paper industry has been associated with viscosity-related issues, such as incomplete mixing [13]. Anaerobic co-digestion of fibre sludge and activated sludge is, therefore, a process where viscosity might play an important role for the process performance, and the anaerobically digested sludge should thus be rheologically characterized.

Other factors that may be affected by fluctuations in the substrates to AD are the microbial formation of extracellular polymeric substances (EPS) and soluble microbial products (SMP) during AD. EPS comprise a wide group of biopolymers mainly consisting of carbohydrates, proteins, humic substances, and lipids (reviewed by [14]). SMP are cellular components that have been released from microbial cells either by diffusion/excretion over the membrane or by cell lysis [15]. The microbial production of EPS is dependent on several parameters, such as the availability of nutrients and changes in temperature and pH [16], while the production of SMP during AD has been shown to increase in response to toxicity, nutrient deficiency or rapid changes in organic loading rate (OLR) [17–19]. Thus, the formation of EPS and SMP is likely sensitive to changes in the substrate composition. The presence of loosely-bound EPS has been shown to decrease the settling ability and dewatering ability of activated sludge as measured by the sludge volume index and specific resistance to filtration [20] and may lead to foaming during AD (reviewed by [21]), which is why increasing the understanding of the causes for its formation is of importance.

Furthermore, there are indications of a relationship between the presence of EPS/SMP and the viscosity of liquids. The importance of EPS in flocculation and biofilm formation is widely recognized (reviewed by [16, 22]), and the functional property of EPS to increase the viscosity in liquids has many industrial applications, for example in dairy products [23] or as a gelling or stabilizing agent in foods or personal care application products [24]. A positive relationship of viscosity to the concentration of EPS has been indicated for activated sludge [25], but if a similar relationship exists in AD sludges has to the authors’ knowledge not yet been shown.

Therefore, an investigation of viscosity and production of EPS and SMP during AD of kraft mill fibre sludge and activated sludge was performed to study how these factors are affected by HRT and/or OLR during long-term AD.

## Materials and methods

### Anaerobic digestion and sludge sampling

Two lab-scale CSTRs (R1 and R2) with working volumes of 4 L were operated for 800 days at 37 °C, as described by [26]. The substrates used were fibre sludge from the primary clarification and activated sludge from the secondary treatment of two kraft pulp and paper mills. To enable the necessary HRT needed for a full-scale AD application, sludge thickening and sludge recirculation were applied. The reactor experiment was divided into four phases: (i) both R1 and R2 digested fibre sludge (day 1–36); (ii) R1 continued to digest fibre sludge, while R2 co-digested fibre sludge and activated sludge (supplied to a TS ratio of 11:1 for fibre sludge to activated sludge), and the OLR was increased stepwise from 0.5 to 3 g VS/L·day (day 37–183); (iii) both reactors co-digested fibre sludge and activated sludge and the OLR was increased temporarily from 3 to 4 g VS/L·day, then back to 3 g VS/L·day (day 284–461); and (iv) the HRT was lowered from 8 to 4 days at maintained OLR, followed by an increase in the OLR from 3 g VS/L·day to 4 g VS/L·day (day 462–800). Every change was first imposed in R2, with R1 acting as a control.

pH was controlled by additions of Ca(OH)_2_, and from day 49, part of the Ca(OH)_2_ was replaced by MgO to increase the concentration of magnesium in the reactors. For details on the feeding procedures, see Ekstrand et al. [26]. Nitrogen and phosphorus were supplied at a ratio of 350:5:1 for COD:N:P, where COD is the chemical oxygen demand of the substrate. Sulphate was added from day 128 at 15 mg/L·day to avoid sulphur deficiency, as indicated by a rapid drop in the levels of H_2_S in the produced biogas from day 100. To investigate if sulphate supplementation could be discontinued, it was decreased stepwise until day 374, after which no sulphate was added. This resulted in heavy foaming, so additions of sulphate were resumed at 10 mg/L·day from day 412.

Sludge was mixed by intermittent stirring using a central 3-bladed pitched-blade impeller (⌀ 70 mm, height 30 mm). Initially, the reactors were mixed 4–5 times a day at 150–400 RPM for 15 min. The frequency and duration were adjusted from day 248 to 20 times a day at 4-min intervals and 400 RPM to avoid fibre accumulation at the surface.

Sludge samples (200 mL) were collected from the reactors once a month for rheological characterization and analysis of EPS and SMP. The samples for rheological measurements were stored at + 4 °C for a maximum of 24 h before rheological characterization. A previous assessment of the effect of overnight sample storage on fluid properties showed no significant changes in the rheological parameters (data not shown). The samples for extraction of EPS/SMP were kept frozen at − 20 °C until time of analysis, when they were thawed at + 4 °C over-night. TS, volatile solids (VS), pH and volatile fatty acids (VFA) were determined as described by [26]. Elemental analysis was carried out once a month for the reactor sludge samples and for every new delivery of substrate by Eurofins Environment Testing Sweden AB.

Correlation analyses were performed in R [27] by calculating Pearson correlation coefficients (*r*). In the cases when the observations were not obtained the same day, they were grouped by week.

The concentration of suspended solids in the water phase (reject water) after centrifugation of the digested reactor sludge was determined in triplicates according to Swedish standard method EN 872-1996 using glass filters (Munktell Glass Microfibre Disc Ø47 mm).

### Rheological characterization

Rheological properties of the digester fluids were determined using a shear rate-controlled Searle-type rotational rheometer (RheolabQC, SN80609650) with a CC27-SN19237 measuring system and a C-LTD80/QC cell coupled with Rheoplus software (Anton Paar, Ostfildern, Germany). The measuring system consisted of a concentric smooth cylinder with an inner diameter of 27 mm, an outer diameter of 29 mm, and a height of 40 mm. Digester samples (19 mL) were analysed in duplicates or triplicates at 37 ± 0.2 °C, corresponding to the operational temperature of the CSTRs. The rheological measurements were made by a modified three-step protocol adapted from [28], where the shear rate was (1) increased linearly from 0 to 800/s over a period of 800 s, (2) maintained constant at 800/s for 300 s, and (3) decreased linearly from 800 to 0/s over a period of 800 s. The corresponding shear stress was registered every 10 s during interval 1 and 3, and every 60 s during interval 2. The certified viscosity reference standard Cannon^©^ RT1000 was used for quality control. Due to the often non-Newtonian character of AD sludge (a non-linear relationship between shear rate and shear stress), apparent viscosity (*η*) was determined for two shear rates, i.e., *η*_100_ at 100/s and *η*_300_ at 300/s. The shear rates were chosen based on the study by Sindall et al. [29], which demonstrated local shear rates of up to 100/s in reactors mixed at 200 RPM, whereas the mixing speed in this study was 150–400 RPM. Shear rates much lower than 100/s showed high variability in the estimated shear stress within the triplicate and were, therefore, not included in the analyses.

### EPS and SMP analyses

EPS and SMP extraction was performed as described by [30], with the following modifications: (i) the reactor sludge samples were diluted in a phosphate buffer solution and centrifuged at 12 000·*g* for 20 min at + 4 °C; (ii) the supernatant was used for SMP analysis, and the pellet was re-suspended in a 200-mL phosphate buffer solution (pH 7) in baffled plastic beakers (500 mL) for EPS extraction; (iii) a cation exchange resin (CER: Dowex^®^ Marathon C, Na^+^ form, Sigma-Aldrich) equal to 80 g CER/g VS was added followed by extraction at 300 RPM on a platform shaker (4 °C) for 18 h and after the extraction, the samples were centrifuged at 12 000·*g* for 20 min.

The concentrations of proteins and polysaccharides were analysed by a modified Lowry method [30] and the Anthrone method [31], respectively, using a UV/VIS spectrophotometer (Ultraspec 2100 pro, Biochrom Ltd, Cambridge, UK). The extracted EPS was quantified as proteinaceous EPS (EPSp) and polysaccharide EPS (EPSc), and the SMP was quantified as proteinaceous SMP (SMPp) and polysaccharide SMP (SMPc). Bovine serum albumin (BSA) and glucose were used as protein and polysaccharide standards, respectively, to determine BSA- and glucose-equivalent concentrations of proteins and polysaccharides in the EPS and SMP fractions. All extractions and analyses were made in triplicates, using three subsamples from each reactor sample.

## Results and discussion

### EPS and SMP

**Fig. 1:**
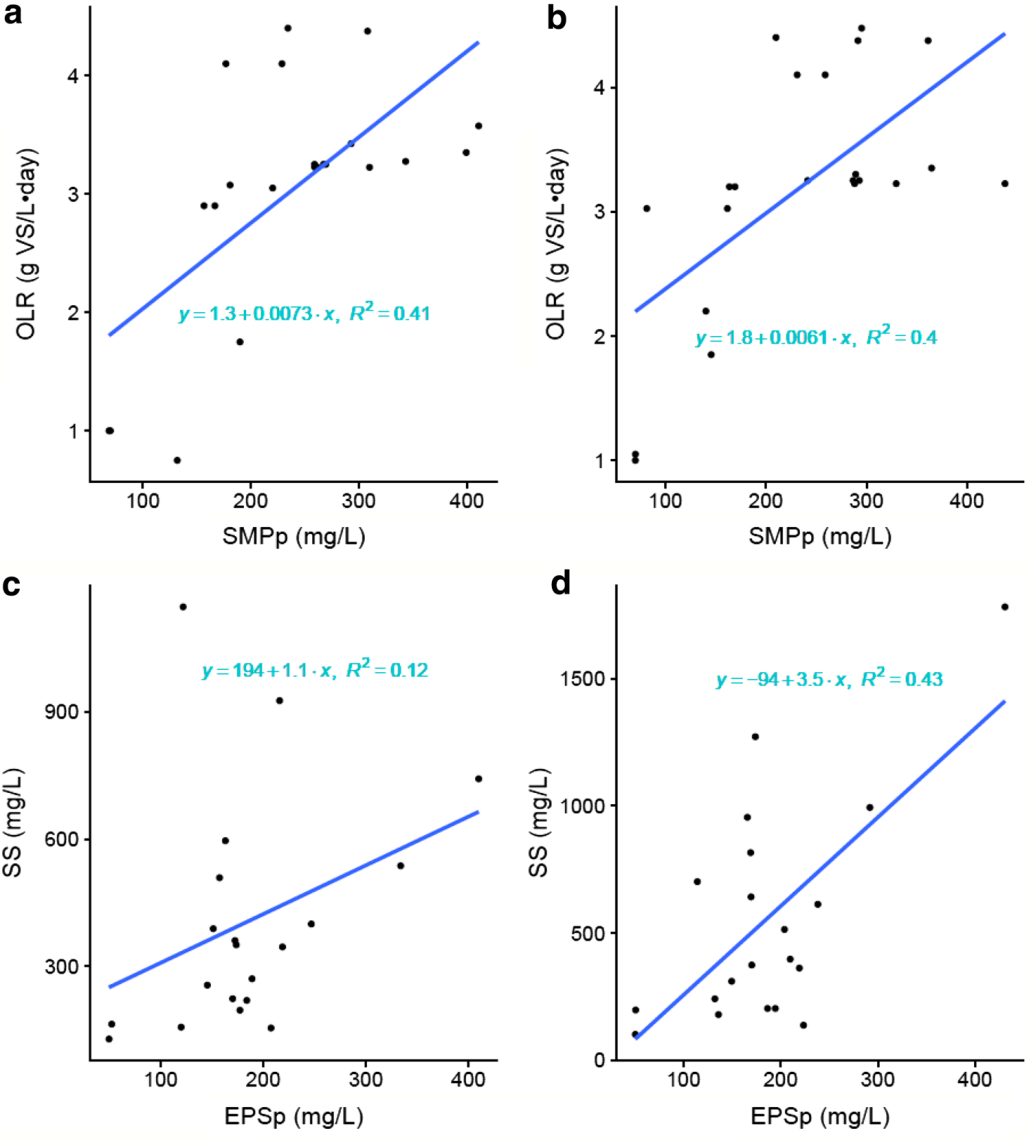
Scatterplots for **a**, **b** the protein fraction of soluble microbial products (SMPp) to the organic loading rate (OLR), measured as volatile solids (VS) and **c**, **d** the protein fraction of extracellular polymeric substances (EPSp) to the amount of suspended solids (SS) in the reject water after centrifugation of the digestate, with reactor 1 (R1) in the left panel and reactor 2 (R2) in the right panel

Of all the investigated parameters, only the production of SMPp was significantly correlated to OLR (Table 1, Fig. 1a, b). This can be an important factor to take into consideration, as SMP are soluble and will lead to reduced treatment efficiencies (i.e., increased COD content in the effluent waters).

**Table 1:** Pearson coefficients for the significant correlations of extracellular polymeric substances (EPS) and soluble microbial products (SMP) to organic loading rate (OLR), hydraulic retention time (HRT), magnesium (Mg) and suspended solids (SS) in the effluent after centrifugation

	EPSp		EPSc		SMPp		SMPc	
	R1	R2	R1	R2	R1	R2	R1	R2
OLR					0.62**	0.62**		
HRT					− 0.54*	− 0.57*		
Mg	0.54**	0.44*	0.47*			− 0.58**	0.66**	0.44*
SS		0.65**		0.76**				0.66**

Changes in OLR and HRT were first implemented in reactor 2 (R2) using reactor 1 (R1) as a control, after which OLR and HRT were also changed in R1. Numbers denoted with * are significant at *p* < 0.05 and ** at *p* < 0.01

**Fig. 2:**
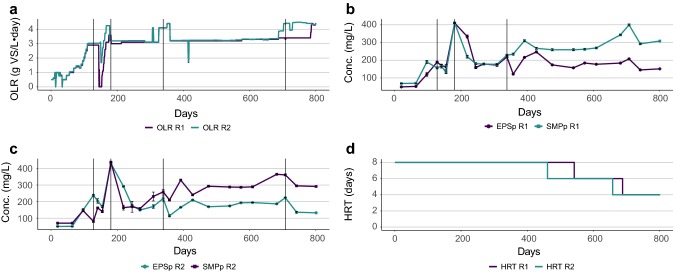
Graph showing **a** the organic loading rate (OLR), **b**, **c** the protein fraction of the extracellular polymeric substances (EPSp) and the protein fraction of the soluble microbial products (SMPp), and **d** the hydraulic retention time (HRT) for reactor R1 and R2 over time (days). The vertical lines marks where the production of EPS and/or SMP increased after the OLR was increased. *Conc*. concentration

**Fig. 3:**
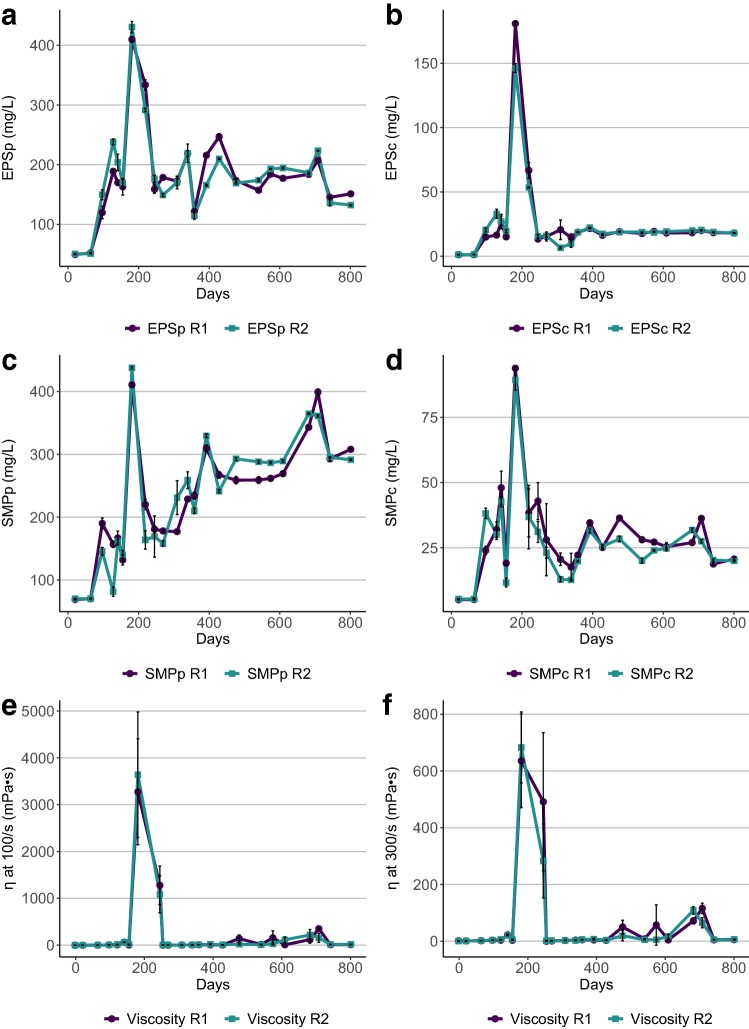
The concentration of extracellular polymeric substances (EPS) and soluble microbial products (SMP) (**a**–**d**) and the corresponding apparent viscosities (**e**, **f**) for reactors R1 and R2, over time (days). The lowercase letters p and s of the EPS and SMP denote the protein and polysaccharide fractions, respectively, and the apparent viscosity was estimated at a shear rate of **e** 100/s and **f** 300/s

EPSp, SMPc, and to some extent EPSc, also responded to changes in OLR, but primarily as peak increases following a change in OLR (Figs. 2a–c, 3b, d). This has been indicated previously in activated sludge processes, where the production of EPS increased temporarily after imposing changes in OLR [20]. Similarly, a temporary increase in production of SMP has been observed after peak additions of glucose in anaerobic chemostats [19], and a study on batch AD of propionate, butyrate and glucose showed a dependency of the EPS production on the food to microorganism ratio (*F*/*M*) [32]. It is possible that when the OLR was first increased and the *F*/*M* ratio was high, the microorganisms responded by increasing the EPS production. Then, as the microorganisms increased in numbers over time due to the higher availability of substrate, the *F*/*M* ratio decreased and the EPS production was halted, giving rise to the temporary increase in EPS which was observed in this study.

Furthermore, the production of SMPp was negatively correlated with HRT (Table 1). The first decrease from an HRT of 8–6 days had a minor impact on the SMPp production only in R2, but after the decrease to 4 days, the level of SMPp increased in both reactors (Fig. 2b–d). For R1, however, the SMPp production had already started to increase before changing the HRT, making the importance of HRT more difficult to evaluate for this reactor. For R2, it was also difficult to separate the effect of the last increase in OLR (day 688–800 for R2) from the last decrease in HRT for R2, as the production in EPS and SMP remained high between the two changes.

Overall, it was primarily changes in OLR that contributed to the EPS and SMP production, showing that this would be the more important factor to control if EPS and SMP production is to be avoided. Though attractive from a methane production perspective, the combination of a high OLR and low HRT would be undesirable from a wastewater treatment perspective, as it led to higher levels SMPp in the effluent.

The concentration of magnesium (mg/kg TS) correlated positively to EPSp, EPSc and SMPc and negatively to SMPp (Table 1). In particular, the concentration of magnesium was high during the peak in EPS and SMP around day 180 (Online Resource 1). The positive effect of divalent cations such as magnesium and calcium on the stability of EPS and its ability to flocculate has been demonstrated in activated sludge [33, 34]. Also, the negative surface charge on the particulate material has been shown to increase with the production of EPS in anaerobic sludge [32], suggesting that the cation bridging seen in EPS in activated sludge could also occur in anaerobic sludge. Furthermore, Mg was supplied at constant levels and the variation of the Mg content of the substrate was small. Therefore, the accumulation of Mg in both reactors showed that Mg was retained in the sludge when the levels of EPS were high, possibly due to negatively charged EPS. Similarly, there was a negative correlation of Mg to SMPp, which could be explained by the mechanism of EPS disintegrating to SMPp. However, more frequent measurements of EPS and SMP content would be required to confirm this mechanism. In summary, the results showed that increased formation of EPS could have more adverse effects on the sludge properties in the presence of Mg.

Dewatering properties of the reactor sludges after AD were not assessed quantitatively in this study, but a decreased separation efficiency during centrifugation was observed when levels of EPS and SMP were high, most notably around days 180 and 390. A positive correlation of EPSp, EPSc and SMPc to the concentration of suspended solids in the effluent was observed for R2 but not for R1 (Table 1, Fig. 1c, d). A possible explanation for the difference could be that activated sludge was added only to R2 during days 37–283, which led to higher concentrations of suspended solids in the effluent of R2 during that period. High levels of suspended solids in the treated water are unwanted, as it leads to poor effluent qualities.

A recognized problem with the extraction of EPS and SMP is that they are both diverse groups of biomolecules and their composition may vary considerably between different microbial populations and environments, and potentially also within the same system. The use of BSA and glucose as standards may lead to overestimations in the amount of proteins and carbohydrates, making the quantification uncertain [35, 36]. However, as this study was performed over a long period of time using the same substrate as a base, the errors are likely to be smaller than if a comparison between different systems would be performed. Yet, the obtained values should be taken as indications of changes and a qualitative assessment of the proteins and carbohydrates present in the system, rather than an exact quantitative determination. Further, the method used for extraction of EPS in this study is more efficient for proteins compared to carbohydrates [30], thus, the true ratio of carbohydrates to proteins is likely underestimated for the sludge. For a more comprehensive extraction of EPS, several different extraction methods may be used, however, the chosen method was suitable to study qualitative changes in EPS over time.

### Viscosity

In general, the rheological characterization of the reactor sludges of R1 and R2 during AD of pulp and paper mill sludges showed low apparent viscosities, i.e., 1–5 mPa·s (Fig. 3e, f). The values were lower than reported apparent viscosities for reactor sludge receiving other types of substrate, e.g., food waste and slaughterhouse waste [9, 37], indicating a lower power consumption for achieving complete mixing compared to the full-scale processes studied by Björn et al., [9]. The flow curves of the two reactors were similar, showing close to Newtonian behaviour with no or low yield stress (not shown). This means that there is minor formation of internal structures when the liquid is at rest, which is why the process may be suitable for intermittent mixing. In addition, the risk of cavern-formation around the impeller and the presence of stagnant zones is low at low yield stress conditions [38].

On some occasions, both reactors experienced an increase in viscosity. This was particularly evident around days 180–250, when the apparent viscosities reached values around 500–700 mPa·s at a shear rate of 300/s. This severely affected the mixing efficiency, which will be discussed more in detail below. Correlation analysis showed, contrary to the literature on the viscosity of activated sludge [5–7], that the increase in viscosity of the reactor sludges during AD of fibre sludge and activated sludge was not correlated with TS. This confirms recent observations that the general relationship between TS and viscosity seen for many types of activated sludge cannot be used to predict the viscosity of all types of AD sludges [9]. Thus, other parameters should be looked for to predict viscosities for AD of sludges from pulp and paper wastewater treatment.

To some extent, the concentrations of EPS and SMP over time seemed positively related to viscosity (Fig. 3). This was in particular indicated around day 180 in both reactors, and around days 708 and 682 for R1 and R2, respectively. As the OLR was increased in the period up to the first peak in viscosity, and HRT was decreased before the peaks around day 700, these factors cannot be excluded as contributors to the increased viscosity. However, a previous study demonstrated a positive relationship between EPS concentration and viscosity for activated sludge [25]. This together with the extensive use of EPS in industrial applications to increase the viscosity in liquids supports that a similar positive relationship may also exist in anaerobic sludge.

### Sludge bulking and foaming

Generally, viscosity and/or the presence of EPS and SMP affected foaming/bulking during AD of fibre sludge by different mechanisms. The drastic increase in viscosity, EPS and SMP during days 170–250 coincided with severe sludge bulking in both reactors, where fibres and produced gas were trapped in the sludge bed, leading to an expanded sludge volume and formation of sludge layers after mixing. To prevent the build-up of gas bubbles and the subsequent formation of bulking layers of fibre and digester sludge during the fast gas release, the stirring frequency and duration were adjusted. Within 2 days, the sludge accumulation on the surface had disappeared. The reason for the particularly high increase in viscosity during this period could be related to the elevated concentration of Mg as discussed above. This explanation is supported by the observation by [39], who demonstrated an elevated viscosity after the addition of EPS to a solution of Mg. Thus, the increase in EPS concentrations occurring in our reactors at elevated Mg concentrations is a plausible explanation for the increase in viscosity in reactor sludges. Another severe foaming event started gradually and culminated during days 390–410 with a frothy, bubbly foam appearing on the liquid surface of the reactors. This coincided with a peak in the concentrations of EPSp, SMPp and SMPc during days 390–430 (Fig. 3a–d) and was likely brought about by removing sulphate from the substrate on day 374. The removal led to extensive foaming in both reactors, but with no effect on the viscosity. Resuming the addition of sulphate resulted in a decrease in the concentrations of EPSp and SMPp and that foaming seized. Likely, the microorganisms suffered from a sulphur deficiency, which induced the excessive production of EPSp and SMPp. This is in line with previous observations on increased production of SMPp after induced nutrient deficiency [17]. During the increased viscosity and sludge-bulking event, there was a clear increase in all fractions of EPS and SMP (Fig. 3), whereas primarily the protein fractions increased during the period of bubbly foaming. This is consistent with the literature on the surface-active properties of proteins and their foaming potential [21]. This difference in EPS and SMP composition could be the reason why there was no effect on viscosity compared to the increased viscosity seen on days 170–250 and around day 700, but more detailed analyses would be required to confirm this. Another explanation could be that much of the EPS and SMP was trapped in the foam on the surface of the reactor sludges and thereby did not lead to structural build-up in the sludge as was seen on days 170–250.

## Conclusions

Our study showed that the production of EPS and SMP and apparent viscosity varied during long-term AD of pulp and paper mill sludge. Specifically, the production of SMPp was positively correlated to OLR, with the implication of reduced effluent qualities at high OLR. From a mill perspective, where treatment efficiency and staying below emission levels is more important than optimizing the methane production, a lower OLR would thus be preferred. Further, EPSp, SMPc, and to some extent EPSc, responded to changes in OLR, but primarily as peak increases following changes in OLR. SMPp was negatively correlated to HRT, but the effect of HRT was not as strong in R1, therefore, this relationship needs further investigation. Magnesium was important for EPS formation, possibly by bridging and strengthening the EPS matrix. It was also demonstrated that sulphur deficiency increased the production of EPS and SMP, and that high levels of EPS and SMP led to poor dewatering during centrifugation, as measured by increased levels of suspended solids in the effluent.

Viscosity was generally low during the experiment, but on some occasions, the viscosity increased in both reactors. The results showed a positive relationship between viscosity and the production of EPS and SMP. Further, the increased viscosity and/or the production of EPS and SMP were important factors in sludge bulking and foam formation. Increased viscosity led to the entrapment of fibres and produced gas in the sludge bed, which was avoided by increasing the mixing frequency. The increased production of EPSp and SMPp induced by the removal of sulphate additions caused heavy foaming on the surface of the reactor sludge. Contrary to results on viscosity measurements in activated sludge, TS did not correlate with viscosity during AD of pulp and paper mill sludge, confirming that solely TS is not enough to predict the viscosity of all types of AD sludge.

Our results imply that viscosity is an important factor for sludge bulking and that rheological analyses may be used to predict and avoid bulking events. In cases when high viscosity leads to entrapment of gas in the sludge bed, a change in the mixing protocol may be a fast way to remedy the situation, decreasing the risk for costly process disruption and equipment damage.

## Electronic supplementary material

Below is the link to the electronic supplementary material.

Supplementary material 1 (DOCX 29 kb)
