# Supplementary material for: Viscosity dynamics and the production of extracellular polymeric substances and soluble microbial products during anaerobic digestion of pulp and paper mill wastewater sludges
Source: Bioprocess Biosyst Eng. 2019 Oct 10;43(2):283–91. doi: 10.1007/s00449-019-02224-4 (PMC6960218; doi:10.1007/s00449-019-02224-4)
Supplement: Supplementary file 1 — Supplementary material 1 (DOCX 29 kb) [file 449_2019_2224_MOESM1_ESM.docx]

# Online Resource 1

Submitted to: Bioprocess and Biosystems Engineering

Article title: Viscosity dynamics and the production of extracellular polymeric substances and soluble microbial products during anaerobic digestion of pulp and paper mill wastewater sludges

Eva-Maria Ekstrand*^1^, Bo H. Svensson^1^, Luka Šafarič^1^ and Annika Björn^1^

^1^ Department of Thematic Studies, Environmental Change, Linköping University, 581 83, Linköping, Sweden

* Corresponding author: [eva-maria.ekstrand@liu.se](mailto:eva-maria.ekstrand@liu.se), +46700-896546, ORCID: 0000-0001-5260-1826

A

B

The concentration of magnesium (Mg) and the protein fraction of extracellular polymeric substances (EPSp) in mg/L for A) reactor R1 and B) reactor R2.
